# Developments in the structural science of materials

**DOI:** 10.1107/S2052252517006145

**Published:** 2017-04-25

**Authors:** C. Richard A. Catlow

**Affiliations:** a Department of Chemistry, University College London, 20 Gordon St., London WC1H OAJ, UK; b School of Chemistry, Cardiff University, Cardiff CF10 3AT, UK

**Keywords:** structural science, materials, computational methods, editorial

## Abstract

Recent developments in the structural science of materials and the growing power of computational methods in this field are discussed.

The papers published during the last year in **IUCrJ** in the fields of materials and computational science illustrate well the challenges posed by structural problems in the science of materials and the key role that computation can play in this and related fields in structural science. As in previous years, they demonstrate the continuing developments in techniques and instrumentation and the increasingly complex structural problems which these developments now make accessible; the role of computation in interpreting and predicting structures is equally clear.

An excellent example of technical developments facilitating new structural science is provided by the article of Meng & Zuo (2016 ▸), which probes three-dimensional nano-structures using a technique that employs high-resolution and low-dose scanning electron nano-diffraction (SEND) to acquire three-dimensional diffraction patterns. Their work investigates TiN – a material that is widely used in the electronics industry – and Fig. 1 ▸ illustrates how they were able to reconstruct grain structures within the material. Detailed knowledge of this microstructure is essential in understanding and optimizing the properties of the material

Previous editorials have emphasized the key role of diffuse scattering, which is also facilitated by technical advances. The importance of the field in materials science is well illustrated by the article of Sawa (2016 ▸), which highlights the work of Welberry & Goossens (2016 ▸) on the interpretation of diffuse scattering from the high-temperature superconductor, HgBa_2_CuO_4 + δ_. Analysis of the diffuse scattering data reveals fascinating features involving the displacement of metal atoms around oxygen interstitial chains. This article along with several others demonstrates the need to elucidate complex structural features in disordered materials.

Analysis of diffuse scattering is also vital in the particularly exciting challenge of developing detailed models for the atomic arrangements in quasicrystals. The article of Ishimasa (2016 ▸) highlights the study of Yamada *et al.* (2016 ▸) on the atomic structure and phason modes of the Sc–Zn icosahedral quasicrystal, which employs synchrotron-based diffraction and diffuse scattering to investigate this difficult problem.

The complexity of structural problem that can now be addressed is well illustrated in the paper of Rozhdestvenskaya *et al.* (2017 ▸), who use a wide range of techniques including several electron crystallographic methods, XRPD and modelling to solve the structure of denisovite, a highly complex, fibrous, polytypical silicate. The structure revealed is shown in Fig. 2 ▸. The article is an elegant illustration of the capacity of, and the need for, a multi-technique approach in addressing structural problems in materials science,

A further example of complex structural science is given by the study of SnTe reported by Sist *et al.* (2016 ▸). This material is increasingly investigated owing to its potential as a thermoelectric material and as a topological insulator. Their study again reveals the importance of disorder and emphasizes the need to include the effects of disorder in any theoretical investigation of the material.

Several papers illustrate both the growing power of computational methods in structural science and the role of new methodologies and algorithms in investigating structural problems. Genoni *et al.* (2017 ▸) explore the concept of X-ray-constrained Hartree–Fock wavefunctions (XC–WF) and discuss how the procedure can be used to extract correlation effects. Their careful analysis demonstrates that the single determinant XC–WF only partially captures the effects of correlation. The paper of Wall (2016 ▸) on quantum crystallography and the charge density of urea shows, as the authors comment, the benefits and feasibility of integrating fully periodic quantum charge-density calculations into ultra-high-resolution X-ray crystallographic model building and refinement. While the value of force-field-based methods is illustrated by the paper of Li *et al.* (2017 ▸), who evaluate different force fields in the context of their use in dynamical simulations for the prediction of chemical shifts in solid-state NMR.

The importance of the structural science of materials is, of course, illustrated by many other articles published in other journals. Of particular interest is the way in which multi-technique approaches are pinning down key structural features of catalytic materials under real operating conditions. We have previously highlighted the work of Lezcano-Gonzalez *et al.* (2016 ▸), which combines high-resolution fluorescence-detection X-ray absorption near-edge spectroscopy, X-ray diffraction and X-ray emission spectroscopy under *operando* conditions to provide detailed new insights into the nature of the Mo species on zeolite ZSM-5 during methane de­hydro­aromatization. Another recent example is the work of Malta *et al.* (2017 ▸), who combined XAFS and modelling to show that in an industrially important acetyl­ene hydro­chlorination catalyst, comprising gold on a carbon support, the active sites are not, as previously thought, gold nano-clusters but single gold ions. Catalysis will unquestionably continue to pose fascinating problems for structural science.

It is hoped that this brief survey gives an impression of the range and excitement of the field of the contemporary structural science of materials and the way in which this can be unravelled by a multi-technique approach using experiment and computation. **IUCrJ** continues to welcome submissions in this growing field.

## Figures and Tables

**Figure 1:**
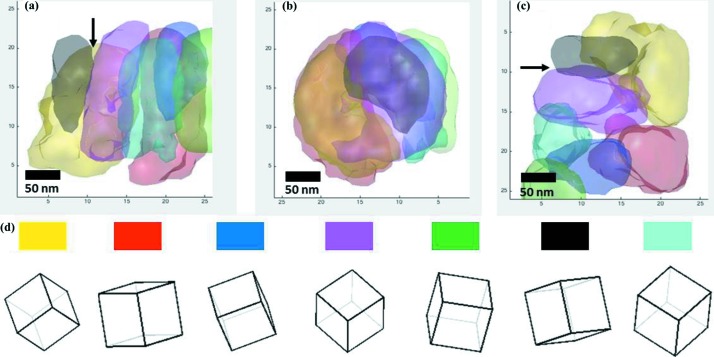
Reconstructed grains and their orientations (Meng & Zuo, 2016 ▸). (*a*) Side view, (*b*) front view and (*c*) top view of the three-dimensional morphologies of the reconstructed grains (a Σ9 grain is indicated by the arrows). (*d*) The orientations of the seven grains. Each cube is labelled by the colour used to represent the grain in parts (*a*)–(*c*).

**Figure 2:**
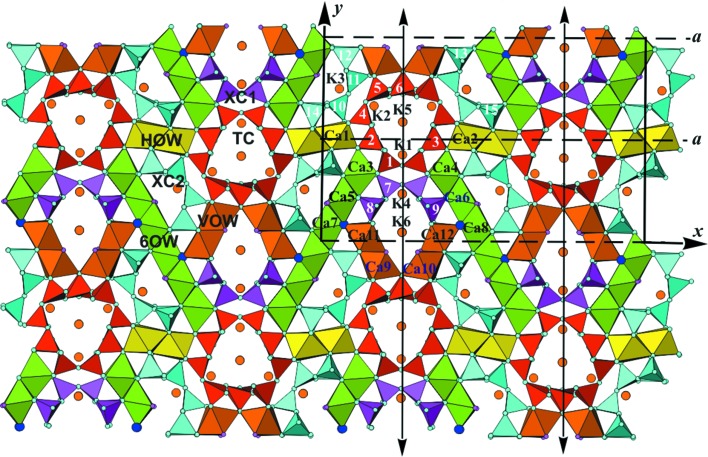
A polyhedral representation of the denisovite structure (Rozhdestvenskaya *et al.*, 2017 ▸) as obtained from the *ab initio* structure solution, shown down the *z* axis. The unit cell and symmetry elements are shown. Tubular loop-branched dreier triple chains [Si_12_O_30_]^12−^ (**TC**) are shown in red, and xonotlite-like dreier double chains [Si_6_O_17_]^10−^ (**XC1**) and (**XC2**) are shown in purple and turquoise, respectively; ‘horizontal’ octahedra walls (**HOW**) are coloured yellow, walls from two (Ca9—C*a*12) octahedra (**VOW**) brown and six-octahedra-wide walls (**6OW**) green. Small turquoise circles are O atoms, purple circles are F atoms, O43 and O44 are marked by blue circles and large brown circles indicate K atoms. Si atoms are numbered.

## References

[bb2] Genoni, A., Dos Santos, L. H. R., Meyer, B. & Macchi, P. (2017). *IUCrJ*, **4**, 136–146. 10.1107/S2052252516019217 PMC5330524 28250952

[bb3] Ishimasa, T. (2016). *IUCrJ*, **3**, 230–231. 10.1107/S2052252516009842 PMC4937777 27437109

[bb4] Lezcano-Gonzalez, I., Oord, R., Rovezzi, M., Glatzel, P., Botchway, S. W., Weckhuysen, B. M. & Beale A. M. (2016) *Angew. Chem. Int. Ed.*, **55**, 5215–5219. 10.1002/anie.201601357 PMC5069576 26990500

[bb5] Li, X., Neumann, M. A. & van de Streek, J. (2017). *IUCrJ*, **4**, 175–184. 10.1107/S2052252517001415 PMC5330528 28250956

[bb6] Malta, G., Kondrat, S. A., Freakley, S. J., Davies, C. J., Lu, L., Dawson, S., Thetford, A., Gibson, E. K., Morgan, D. J., Jones, W., Wells, P. P., Johnston, P., Catlow, C. R. A., Kiely, C. J. & Hutchings, G. J. (2017). *Science*, **355**, 1399–1403. 10.1126/science.aal3439 28360324

[bb7] Meng, Y. & Zuo, J.-M. (2016). *IUCrJ*, **3**, 300–308. 10.1107/S205225251600943X PMC5391852 28461891

[bb8] Rozhdestvenskaya, I. V., Mugnaioli, E., Schowalter, M., Schmidt, M. U., Czank, M., Depmeier, W. & Rosenauer, A. (2017). *IUCrJ*, **4**, XXX–XXX. 10.1107/S2052252517002585 PMC5414397 28512570

[bb9] Sawa, H. (2016). *IUCrJ*, **3**, 298–299. 10.1107/S2052252516013889 PMC5391851 28461890

[bb10] Sist, M., Jensen Hedegaard, E. M., Christensen, S., Bindzus, N., Fischer, K. F. F., Kasai, H., Sugimoto, K. & Brummerstedt Iversen, B. (2016). *IUCrJ*, **3**, 377–388. 10.1107/S2052252516012707 PMC5391859 28461898

[bb11] Wall, M. E. (2016). *IUCrJ*, **3**, 237–246. 10.1107/S2052252516006242 PMC4937779 27437111

[bb12] Welberry, T. R. & Goossens, D. J. (2016). *IUCrJ*, **3**, 309–318. 10.1107/S2052252516010629 PMC5391853 28461892

[bb13] Yamada, T., Takakura, H., Euchner, H., Pay Gómez, C., Bosak, A., Fertey, P. & de Boissieu, M. (2016). *IUCrJ*, **3**, 247–258. 10.1107/S2052252516007041 PMC4937780 27437112

